# 5-Azacytidine Induces Anoikis, Inhibits Mammosphere Formation and Reduces Metalloproteinase 9 Activity in MCF-7 Human Breast Cancer Cells

**DOI:** 10.3390/molecules19033149

**Published:** 2014-03-13

**Authors:** Hsueh-Wei Chang, Hui-Chun Wang, Chiau-Yi Chen, Ting-Wei Hung, Ming-Feng Hou, Shyng-Shiou F. Yuan, Chih-Jen Huang, Chao-Neng Tseng

**Affiliations:** 1 Department of Biomedical Science and Environmental Biology, Translational Research Center, Kaohsiung Medical University Hospital, Kaohsiung Medical University, Kaohsiung 80708, Taiwan; E-Mail: changhw@kmu.edu.tw; 2 Institute of Medical Science and Technology, National Sun Yat-Sen University, Kaohsiung 80424, Taiwan; 3 Graduate Institute of Natural Products, Kaohsiung Medical University, Kaohsiung 80708, Taiwan; E-Mails: wanghc@kmu.edu.tw (H.-C.W.); ionemicky@hotmail.com (C.-Y.C.); kva916@yahoo.com.tw (T.-W.H.); 4 Cancer Center and Department of Surgery, Kaohsiung Medical University Hospital, Kaohsiung 80708, Taiwan; E-Mail: mifeho@kmu.edu.tw; 5 Translational Research Center, Department of Obstetrics and Gynecology, Kaohsiung Medical University Hospital, Kaohsiung 80708, Taiwan; E-Mail: 1020005@kmuh.org.tw; 6 Department of Radiation Oncology, Kaohsiung Medical University Hospital, Kaohsiung 80708, Taiwan; 7 Faculty of Medicine, College of Medicine, Kaohsiung Medical University, Kaohsiung 80708, Taiwan; 8 Department of Biological Sciences, National Sun Yat-sen University, Kaohsiung 80424, Taiwan

**Keywords:** cancer stem cell, breast cancer, anoikis

## Abstract

Cancer stem cells are a subset of cancer cells that initiate the growth of tumors. Low levels of cancer stem cells also exist in established cancer cell lines, and can be enriched in serum-free tumorsphere cultures. Since cancer stem cells have been reported to be resilient to common chemotherapeutic drugs in comparison to regular cancer cells, screening for compounds selectively targeting cancer stem cells may provide an effective therapeutic strategy. We found that 5-azacytidine (5-AzaC) selectively induced anoikis of MCF-7 in suspension cultures with an EC_50_ of 8.014 µM, and effectively inhibited tumorsphere formation, as well as the migration and matrix metalloproteinases-9 (MMP-9) activity of MCF-7 cells. Furthermore, 5-AzaC and radiation collaboratively inhibited MCF-7 tumorsphere formation at clinically relevant radiation doses. Investigating the underlying mechanism may provide insight into signaling pathways crucial for cancer stem cell survival and pave the way to novel potential therapeutic targets.

## 1. Introduction

Most types of cancer, including breast cancer, originate from a small population of cancer stem cells (CSCs) [1,2,3,4]. These CSCs are able to self-renew and give rise to other cancer cells that form a tumor mass [5,6]. Breast cancer stem cells can be established from patients’ surgical specimens, based on their ability to resist cell-detachment-induced apoptosis (anoikis) and to propagate *in vitro* as floating tumorspheres in suspension cultures [7]. Tumorspheres show an increase in CSC population, overexpress neoangiogenic and cytoprotective factors, and display high tumorigenic potential in NOD/SCID mice [7,8]. Established breast cancer cell lines also contain a small percentage of cancer stem cells that can be enriched in tumorsphere cultures [9,10]. Therefore, suspension cultures of breast cancer cell lines have been used as a drug screening platform, and a number of reagents that target CSCs have been successfully identified [11,12]. 

CSCs have been implicated in the resistance of cancer to conventional chemotherapy [13,14], and likely play an essential role in metastasis [15]. In addition, CSCs are relatively radioresistant, likely due to their heightened DNA repair [16] and free-radical scavenging abilities [17]. Conversely, radiation has been found to increase matrix metalloproteinases expression as well as migration and invasion in various cancer cell lines, including MCF-7 and MDA-MB-231 [18,19,20,21]. 

5-Azacytidine (5-AzaC) and 5-aza-2'-deoxycytidine (5-AzadC) are nucleoside analogues designed to reduce DNA methylation and have been used clinically for treating acute myelogenous leukemia [22,23]. These cytidine analogues have diverse but overlapping effects on gene expression [24], and on cellular survival [25]. 5-AzaC has also been found to enhance the reprogramming efficiency of murine induced pluripotent stem cells by activating the expression of dormant genes [26,27]. However, the effects of 5-AzaC on breast cancer stem cells have not been reported. 

## 2. Results and Discussion

### 2.1. 5-Azacytidine Sensitizes MCF-7 Cells to Anoikis

**Figure 1:**
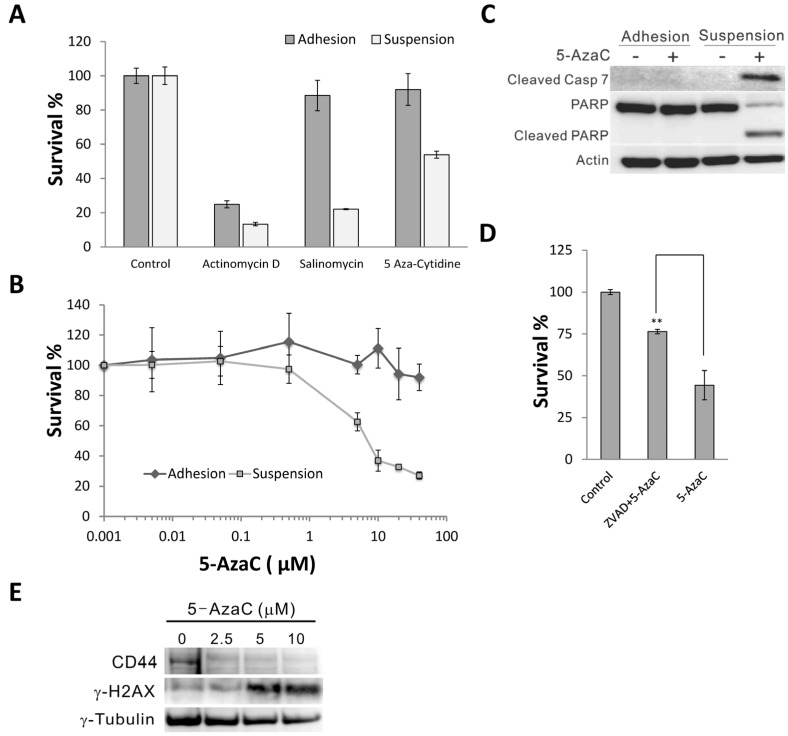
(**A**) Effects of 5 μM actinomycin D, salinomycin, and 5-AzaC on the survival of MCF7 in attachment and suspension cultures (48 h). (**B**) 48 h survival curves of MCF-7 attachment and suspension cultures treated with 5-AzaC. (**C**) 5-AzaC (10 μM, 24 h) selectively induced the cleavage of caspase 7 and PARP in suspension MCF7 cells as determined by western blotting. (**D**) Pretreatment of 10 μM Z-VAD-fmk for 2 h increased the survival of MCF7 suspension cultures treated with 10 μM 5-AzaC for 48 h. (**E**) Expression of CD44 and γ-H2AX in MCF7 suspension cultures treated with 0–10 μM 5-AzaC for 24 h.

To test the effects of 5-AzaC on the anoikis resistance of MCF-7 human breast cancer stem cells, we first examined the 48 h survival of MCF-7 suspension cells in the presence of 5 μM 5-AzaC. Equimolar amounts of actinomycin D and salinomycin [11] served as the control for non-discriminatory cytotoxic agent and selective cancer stem cell inhibitor, respectively. Like salinomycin, 5-AzaC displayed selective toxicity toward suspended MCF-7 cells (Figure 1A). The dose-response study further confirmed the selective toxicity of 5-AzaC toward suspended cells, even at 50 μM (Figure 1B). EC_50_ was determined to be 8.014 μM using GraphPad Prism. The selective toxicity was due to the induction of anoikis, as 10 μM 5-AzaC induced the activation of caspase 7 and the degradation of poly ADP-ribose polymerase (PARP), and pan-caspase inhibitor Z-VAD-fmk significantly increased the survival of MCF-7 suspension cells treated with 5-AzaC (Figure 1C,D). In addition western blotting indicated that treatment of 5-AzaC for 24 h reduced the expression of breast stem cell maker CD44 and increased the expression of γ-H2AX, an indicator of DNA strand break in MCF7 suspension cultures (Figure 1E). 

### 2.2. 5-AzaC Reduces the Clonogenicity of MCF-7 Cells

**Figure 2:**
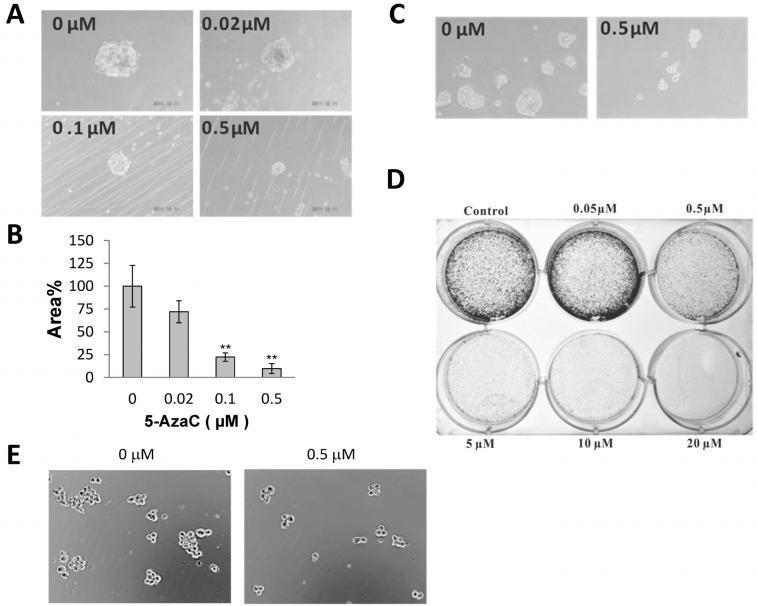
(**A**) Representative microphotographs of MCF-7 mammospheres grown in suspension culture with 0–0.5 μM 5-AzaC for 7 days. (**B**) The areas occupied by mammospheres from the digital microphotographs of 10 random visual fields were determined by ImageJ. Mean ± SD, *n* = 3; ** *p* < 0.01 between control tumorspheres and the ones treated with 5-AzaC. (**C**) Representative microphotographs showing that 0.5 μM 5-AzaC reduced the size of 14 days MCF-7 3-dimentional colonies in soft agar assay. (**D**) 2-dimentional colony formation by adherent MCF-7 cells pre-treated with 0.05–20 μM 5-AzaC for 1 day. Each well was seeded with 1,000 viable cells which were cultured in the absence of 5-AzaC for two weeks, and then the colonies were visualized by crystal violet staining. (**E**) Inhibition of tumorsphere formation in 4 day T47D suspension cultures by 0.5 μM 5-AzaC.

To determine if 5-AzaC inhibits MCF-7 CSC’s ability to repopulate from single cells, we tested the effects of 5-AzaC on MCF-7 colony formation in 3-dimentional and monolayer culture conditions. 5-AzaC, as low as 0.1 μM, effectively inhibited the growth MCF-7 tumorspheres in suspension cultures (Figure 2A,B). 0.5 μM 5-AzaC also reduced the size of MCF-7 colonies embedded in soft agar (Figure 2C). Pretreatment of MCF-7 cells with 5-AzaC at concentrations higher than 0.5 μM for 24 h also reduced the clonal survival of MCF-7 cells in monolayer cultures (Figure 2D). 5-AzaC also inhibited tumorsphere formation of another breast cancer cell line T47D (Figure 2E).

### 2.3. 5-AzaC Inhibits the Migration and Metalloproteinase 9 Activity of MCF-7 Cells

**Figure 3:**
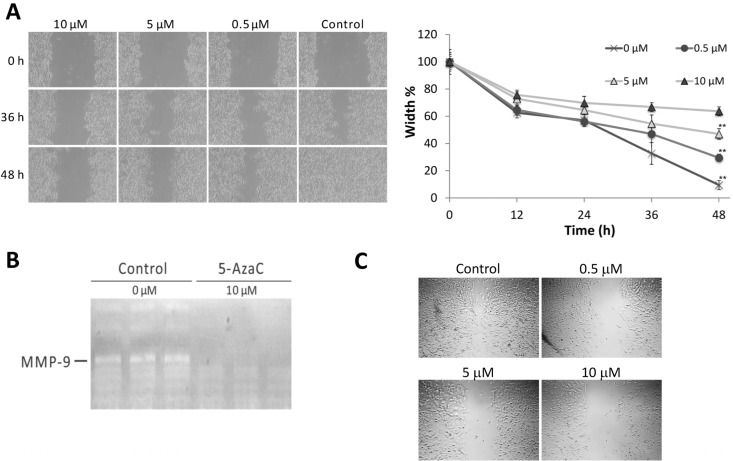
(**A**) Wound healing assay showing the inhibitory effect of 0.5–10 μM 5-AzaC on MCF-7 migration for 48 h. Mean ± SD, *n* = 3; ** *p* < 0.01 between control and cells treated with 5-AzaC. (**B**) Metalloproteinase 9 activities in the supernatant of adherent MCF-7 cultures treated with 0 or 10 μM 5-AzaC for 24 h were determined by gelatin zymography. Results of triplicate experiments are shown. (**C**) Wound healing assay showing the inhibitory effect of 0.5–10 μM 5-AzaC on MDA-MB-231 migration for 36 h.

Previous reports have associated CSC properties with a strong propensity for tumor metastasis [28], and increased cell mobility [29]. We therefore tested the effects of 5-AzaC on the migration ability of and Metalloproteinase 9 (MMP-9) secretion in MCF-7 cells. 5-AzaC higher than 0.5 μM significantly inhibited the gap closure in the wound healing assay (Figure 3A). The activity of MMP9 in the supernatant of adherent MCF-7 cultures was also effectively inhibited by 10 μM 5-AzaC (Figure 3B). 5-AzaC also inhibited the migration of a more aggressive breast cancer cell line, MDA-MB-231 (Figure 3C).

### 2.4. 5-AzadC Is Not Effective in Inducing MCF-7 Anoikis

**Figure 4:**
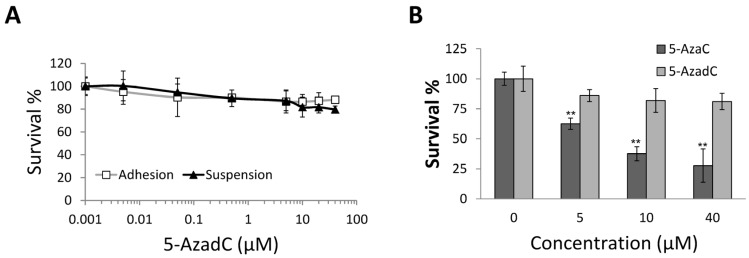
(**A**) 5-Azadeoxycytidine was not cytotoxic to either adhesion or suspension MCF-7 cells. (**B**) Comparison of the effects of 5-AzaC and 5-AzadC on the 48 h survival of suspension MCF-7 cells. Mean ± SD, *n* = 3; ** *p* < 0.01 between cells treated with 5-AzaC or 5-AzadC.

The effects of 5-AzadC, another cytidine analogue that can reduce DNA methylation, on MCF-7 anoikis was also tested and compared to that of 5-AzaC. 5-AzadC displayed very low cytotoxicity toward adhesion or suspension MCF-7 (Figure 4A,B). This suggests that the mechanisms involved in 5-AzaC’s anti-cancer stem cell effects differ from 5-AzadC’s.

### 2.5. 5-AzaC and Radiation Therapy Collaboratively Reduced the Growth of MCF-7 Tumorspheres

**Figure 5:**
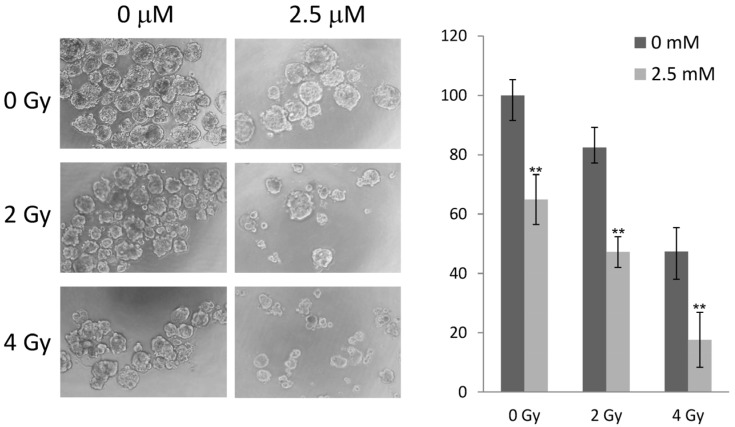
5-AzaC and radiation therapy collaboratively reduced the growth of MCF-7 tumorspheres. MCF-7 adhesion cultures were pretreated with 2.5 μM 5-AzaC for 1 day, irradiated with 0–4 Gy radiation, and then reseeded at 1000 cells/well in an ultralow attachment 96 well plate to allow tumorsphere formation for two weeks (left panel). Percent growth of tumorsphere was determined by WST-1 reagent (right panel). Mean ±SD, *n* = 3; ** *p* < 0.01 between tumorspheres grown with or without 2.5 μM 5-AzaC.

Since CSCs have been known to be more resistant to radiation, we further tested whether pretreating MCF-7 cells with 5-AzaC can sensitize MCF-7 CSCs to radiation with clinically relevant doses. MCF-7 tumorspheres were relatively resistant to 2 Gy radiation but showed reductions in number and size at 4 Gy, whereas MCF-7 cells pretreated with 5 μM 5-AzaC further showed significant reduction in tumorsphere growth (Figure 5).

In summary, we have demonstrated the effectiveness of 5-AzaC in suppressing the survival and growth of MCF-7 cancer stem cells in suspension cultures. This activity is not possessed by 5-AzadC, indicating the underlying mechanism may not be simply explained by global DNA demethylation. Interestingly, 5-AzaC significantly inhibited the clonogenicity and migration abilities of MCF-7 at 0.5 μM, much lower than needed to induce MCF-7 anoikis. This may suggest that 5-AzaC exerts its function by activating more than one signaling pathway. 

## 3. Experimental

### 3.1. Cell Culture

Breast cancer cells were cultured in Dulbecco’s modified Eagle medium (DMEM) supplemented with 10% heat-inactivated fetal calf serum (FCS) and 0.1 mM nonessential amino acids at 37 °C in a humidified atmosphere (5% CO_2_). To culture CSCs and to propagate them as tumospheres, cells were suspended in DMEM–F12 supplemented with 0.4% bovine serum albumin, 5 μg/mL bovine insulin, 20 ng/mL basic fibroblast growth factor 2 (bFGF), and 10 ng/mL epidermal growth factor (EGF) at a density of 1,000 cells/mL in ultralow attachment 96 well plates. Fresh media were replenished every 3 days. 

### 3.2. Assessment of Cellular Viability by Trypan Blue

Viability of dissociated cells was determined using a hemocytometer and the trypan blue dye exclusion method (final dye concentration: 0.04% *w/v*) [30].

### 3.3. WST-1 Assay

WST-1 solution (Roche, Mannheim, Germany) was added to cells at 10 μL/100 μL medium and incubated for 4 h. The absorbance at 450 nm was measured for each well and the average reading of control cells was taken as 100% for normalization. 

### 3.4. Tumorsphere Formation Assay

As described previously [12], the ability of cells of monolayer cultures to initiate tumorsphere formation were assessed by harvesting, washing, and resuspending monolayer cells in cancer stem cell medium (DMEM–F12 supplemented with 0.4% BSA, 20 ng/mL bFGF, 10 ng/mL EGF and 5 μg/mL insulin). Cells were then passed through a 40-μM sieve, counted, diluted, and seeded into 96-well ultralow attachment plates at 1,000 cells/well. 

### 3.5. Soft Agar Colony Formation Assay

5 × 10^4^ cells were mixed with 2% molten low melting agarose in DMEM–F12 growth medium for a final concentration of 0.4% agarose. The cell mixture was placed on top of a solidified layer of 0.5% agarose with 1 × growth medium. Cells were fed every 6 to 7 days with growth medium [12].

### 3.6. Clonogenic Assay

After 5-AzaC pretreatment, the cells were reseeded at a density of 1 × 10^3^ cells per well in the 6-well culture plate and cultured for 10 to 15 days, with medium changed every 3 days. The colonies were then fixed with methanol-acetic acid (3:1) and stained with 1% crystal violet for 30 min at room temperature. 

### 3.7. Wound-Healing Motility Assay

Cells seeded onto six-well plates were allowed to grow to confluence before scratch wounds were created in each well using a p10 micropipette tip. The cells were washed three times with phosphate-buffered saline (PBS) and incubated with complete medium. Images of wound healing were captured by phase-contrast microscopy at indicated times after wounding. 

### 3.8. Gelatin Zymography

Supernatants from cell cultures were collected, filtered through a 0.22 μM filter, and concentrated 50 × by using Centricon filters with 10 kD cutoff. Concentrated supernatants were denatured in 1 × non-reducing SDS gel sample buffer and applied without boiling to a 10% polyacrylamide gel containing 0.1% SDS and 1 mg/mL gelatin. After electrophoresis, the gels were washed in 50 mM Tris-HCl (pH 7.5) containing 0.15 M NaCl, 5 mM CaC1_2_, 5 μM ZnCl, 0.02% NaN_3_, 0.25% Triton X-100 at room temperature for 30 min three times, and then incubated in the same buffer without Triton X-100 at 37 °C for 20 h. The gelatin-clear zones were visualized by staining with Coomassie Brilliant Blue R-250 solution.

### 3.9. Western Blotting

Western blotting was performed as described before [31]. Antibodies used in this study are: Cleaved Caspase-7 (Asp198) (D6H1) Rabbit mAb #8438, PARP Antibody #9542, Cleaved PARP (Asp214) (D64E10) XP^®^ Rabbit mAb #5625 from Cell Signaling Technology (Danvers, MA, USA); Anti-phospho-Histone H2A.X (Ser139) Antibody, clone JBW301 and Anti-CD44 [H-CAM] Antibody, clone EPR1013Y from Millipore (Billerica, MA, USA); γ-Tubulin Antibody (4D11) and Beta Actin Loading Control Antibody (BA3R) from ThermoFisher Scientific (Waltham, MA, USA).

### 3.10. Statistical Analysis

All experiments were repeated at least three times. Results are shown as the mean ± SD from three independent experiments. Data were analyzed by Student’s T-test. *p* values < 0.05 were considered statistically significant. Single and double asterisks indicate *p* < 0.05 and *p* < 0.01, respectively.

## 4. Conclusions

Our results show that 5-AzaC effectively suppressed multiple aspects of MCF-7 human breast cancer stem cell activities: anoikis resistance, tumorsphere formation, and cellular migration capabilities. 
